# Ethics, patient rights and staff attitudes in Shanghai's psychiatric hospitals

**DOI:** 10.1186/1472-6939-13-8

**Published:** 2012-05-17

**Authors:** Liang Su, Jingjing Huang, Weimin Yang, Huafang Li, Yifeng Shen, Yifeng Xu

**Affiliations:** 1 Department of Psychiatry, Huashan Hospital, Fudan University, School of Medicine, No. 12 Wulumuqi Road (middle), Shanghai, 200040, People's Republic of China; 2 Shanghai Mental Health Center, Shanghai Jiao Tong University School of Medicine, No. 600 Wan Ping Nan Road, Shanghai, 200030, People's Republic of China

**Keywords:** Psychiatric hospital, Bioethics, Hospital Ethics Committees, Health Care Surveys, China

## Abstract

**Background:**

Adherence to ethical principles in clinical research and practice is becoming topical issue in China, where the prevalence of mental illness is rising, but treatment facilities remain underdeveloped. This paper reports on a study aiming to understand the ethical knowledge and attitudes of Chinese mental health professionals in relation to the process of diagnosis and treatment, informed consent, and privacy protection in clinical trials.

**Methods:**

A self-administered survey was completed by 1110 medical staff recruited from Shanghai’s 22 psychiatric hospitals. Simple random selection methods were used to identify target individuals from the computerized registry of staff.

**Results:**

The final sample for analysis consisted 1094 medical staff (including 523 doctors, 542 nurses, 8 pharmacologists and 21 other staff). The majority reported that their medical institutions had not established an Ethics Committee (87.8%) and agreed that Ethics Committees should be set up in their institutions (72.9%). Approximately half (52%) had not received systematic education in ethics, and almost all (89.1%) of the staff thought it was necessary. Nearly all participants (90.0%) knew the Shanghai Mental Health Regulations which was the first local regulations relating to mental health in China, but only 11% and 16.6% respectively knew of the Nuremberg Code and the Declaration of Helsinki. About half (51.8%) thought that the guardian should make the decision as to whether the patient participated in clinical trials or not.

**Conclusions:**

The study indicates that most psychiatric hospitals in Shanghai have no Medical Ethics Committee. More than half the medical staff had not received systematic education and training in medical ethics and they have insufficient knowledge of the ethical issues related to clinical practice and trials. Training in ethics is recommended for medical staff during their training and as ongoing professional development.

## Background

Social changes that are in progress in China have contributed to a high prevalence of mental illness with more than 100 million people now having a mental disorder, and about 16 million of these having a serious mental illness [1]. The burden of mental illnesses in low-income and middle-income countries is estimated to be three-quarters of the global burden for these conditions [2]. In middle-income countries (including China)—where the disease burden attributable to these conditions exceeds that for infectious, cardiovascular, or neoplastic diseases—neuropsychiatric disorders are already the major causes of illness in both men and women. However, treatment for these disorders is inadequate. In China, for example, psychiatric institutions have insufficient facilities or are inadequately equipped to treat people with mental disorders, who are often highly stigmatized [3].

The potentially easy access to large samples of psychiatrically ill people makes China an ideal place to conduct research into mental illnesses. While in the past there may have been few barriers to such research, over the last two decades medical research ethics has been a growing concern [4]. In the 1990s, ethical guidelines from the Western world were translated into Chinese, and many ethics workshops were conducted in major Chinese research centers [5]. Since then, increased access to worldwide media, exposure to western value systems, and participation in national and international web-based networking have contributed to wide-ranging challenges to traditional values and cultural practices [6]. For example there have been criticisms of the principle-based framework in China [7], in which the traditional ethic emphasizes social harmony over individual interests. These traditional Chinese ethics focus on the responsibility of a person to work for the good of others, rather than on adherence to general principles of common morality as seen in the virtue-based ethics of developed countries [8].

In parallel to the challenges to traditional ethical principles in China, in recent years, economic incentives in medical practice and research have exacerbated the potential for conflicts of interest [9], which can undermine ethical relations between medical professionals and researchers, drug companies or other research funders [10], patients who become research participants, and even the regulatory agencies whose mandate is to protect participants’ safety [11]. Of particular concern is that there is lack of ethics information for medical staff who conduct research in psychiatric hospitals, where patients are particularly vulnerable [12]. The main objective of this study is to understand the ethical attitudes of mental health professionals in Shanghai in relation to the process of diagnosis and treatment, informed consent, and privacy protection, in order to facilitate the improvement of compliance with ethics principles in psychiatric institutions in China.

## Methods

### Sample selection and screening and research sites and sampling procedures

**Figure 1:**
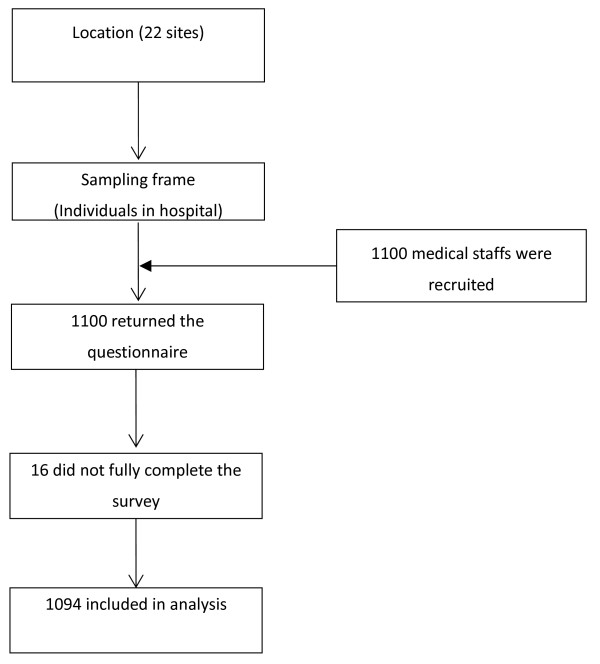
Research sites and sampling selection procedures and screening.

Shanghai has a population of 19,213,200 and the GDP per capita in Shanghai is over US$ 4,500. There are 22 psychiatric hospitals in Shanghai, all of which were included in the study. At each site, simple random selection methods were used to identify target individuals from the computerized registry of staff in the hospital (Figure 1). In each hospital, 50 staff were randomly selected, except at two sites where 55 staff were selected because these two hospitals have more staff. This resulted in a sample of 1110 medical and health service professionals, including 523 doctors, 542 nurses, 8 pharmacologists and 21 other staff.

Instruments: A questionnaire survey was used for the study. We developed the questionnaire based on the World Health Organization guidelines for committees reviewing research, related regulations in China such as *Approaches to Ethics Review of Biomedical Research Involving Human Subjects* (Draft) (Ministry of Health) and *Good Clinical Practice for Drugs* (State Food and Drug Administration). We adjusted initial versions of the questionnaires according to a pilot study and consultation among experts. The instrument (in Chinese) was designed to be self-administered.

The questionnaire consisted of 6 sections with a total of 80 questions, covering general demographic information such as age and years working, the site of work, education relating to medical ethics, work conditions, attitudes relating to diagnosis and treatment, informed consent, and privacy. The questionnaire had single-choice, multiple-choice and open-ended questions. If the respondent did not agree with any of the available options, she or he could write their opinion on the survey form. For example, one question asked: "Do you agree that patients must give written informed consent when they participate in clinical trials?" The respondent could choose: 1 for completely disagree, 2 for disagree, 3 for neither agree nor disagree, 4 for agree, 5 for fully agree, and 0 for not applicable, or other response which was then elaborated by the respondent. The participants were also surveyed in relation to their self-assessment of the knowledge of the process of informed consent, and their opinions and suggestions on related issues.

The data were collected from October to December 2010. Of the 1100 distributed survey questionnaires, 1100 were returned (response rate of 100%).^1^There was no economic incentive to complete the survey. Sixteen staff returned incomplete surveys (missing items more than 5%), and their data were excluded from analyses, leaving 1094 (98.56%) cases for statistical analysis.

The Medical Ethics Committee of Shanghai Mental Health Center provided ethics approval for the study. Respondents provided oral informed consent to participate in the study before the completing the questionnaire.

### Statistical analysis

Data collected were entered into Epidata, version 3.1 (EpiData Association, Odense, Denmark), and Microsoft Excel 2003. SPSS 15.0 (Statistical Package for the Social Sciences Company, Chicago, IL) was used to derive descriptive statistics. Frequencies and corresponding percentages for dichotomous variables or medians and interquartile ranges for continuous variables were calculated. The qualitative data were summarized by theme.

### Role of the funding source

The sponsor had no role in the design, execution, data analysis, or writing up of the study. The corresponding author had full access to all study data and had final responsibility for the decision to submit the paper for publication.

## Results

Nearly all of the participants (97.1%) were drawn from secondary psychiatric hospitals which have approximately 300 beds, with the remainder drawn from tertiary psychiatric hospitals which have around one thousand beds and deal with more severe cases. Demographic characteristics (Table 1) showed the sample captured a range of experience of staff, with the mean length of service (ranging from 6 months to 32 years) being 12.6 years. Participants were aged between 19 and 62 (mean = 34.56) years, and 74.45% of the sample was female. The majority of the participants (98.45%) were Han Chinese, were married (69.7%), had completed high school or university (99.3%) and had no religion (96.4%).

**Table 1:** Demographic characteristics of medical staffs in psychiatric hospitals in Shanghai

**Characteristic**	**Respondents (n=1094) N (%)**	**Missing n (%)**
Age in years (Mean ± S.D.)	34.56±10.13	6 (0.55%)
Working Experience (years)	12.60±10.29	1 (0.091%)
Sex		0
Male	280(25.6%)	
Female	814(74.45%)	
Nationality		4 (0.37%)
Han	1077(98.45%)	
Others (Hui and Manchu)	13(1.2%)	
Marital status		2 (0.18%)
Single	305(27.9%)	
Married	762(69.7%)	
Divorced or Widowed	25(2.3%)	
Professional		0
Doctor	523(47.8%)	
Nurse	542(49.5%)	
Pharmacologist	8(0.7%)	
Others (Care Workers, etc.)	21(2.0%)	
Religion		2 (0.18%)
Roman Catholic	6(0.5%)	
Islam	1(0.1%)	
Other Christian	6(0.5%)	
Buddhism	16(1.5%)	
None	1055(96.4%)	
Education*		0
High	20(1.8%)	
Upper	417(38.1%)	
Middle	650(59.4%)	
Low	7(0.7%)	

*Low, Middle, Upper and High Education levels refer respectively to junior high school and below, high school, college and university undergraduate, and Master’s Degree and above.

### General results

Most of staff (87.8%) reported that their medical institutions had not established an Ethics Committee, and 72.9% of them thought that an Ethics Committee should be set up in their institution. Furthermore, 569 (52%) of the 1094 participants had not received systematic education and training in medical ethics since they commenced working in the field, but 89.1% thought that medical ethics education and training were necessary for all medical staff. Nearly all (97.2%) of the participants reported they had not served as members of an ethics committee that reviewed research proposals. Further, only 4.5% of staff had applied for ethics review for their research projects, clinical trials or new technology projects, and 96.8% had not applied for ethics review when they published research papers.

When it came to knowledge of ethics regulations, 11% and 16.6% respectively of participants knew of the Nuremberg Code and the Declaration of Helsinki. Slightly more, 17.1% and 25.9% respectively, knew the regulations set up by the China State Food and Drug Administration, the "biomedical research involving human subjects ethics review methods" and the "drug clinical trial quality management practices". However, nearly all of them (90.0%) knew Shanghai Mental Health Regulations which was the first set of local regulation related to mental illness on the Chinese mainland, implemented in late 2001. Most participants (83.4%) had not talked with patients or their guardians in relation to patients' participation in clinical trials, only 5.2% of them did not want to interview patients to invite them to participate in clinical trials when asked whether they were willing to do so, and 20.8% of them considered doing so later. The main reason for not being willing to talk with patients (75.57%) were that the medical staff worry about poor efficacy and the side effects of testing drugs in clinical trials. Only 9.7% of the staff thought treatment would be influenced if the patients refused to participate in clinical trials.

### Ethical attitude in the process of diagnosis and treatment, informed consent and privacy protection

Most participants (78.6%) were not sure whether it is helpful for treatment if patients decide to participate in clinical trials. Less than half (41.2%) thought that the relationship between the clinical trials and pharmaceutical companies is too close, and about half (51.8%) thought that the guardian should make the decision whether the patient should participate in clinical trials or not. However, two-thirds (66.9%) thought it is better if patients with mental illness provide informed consent in writing themselves. Other ethics attitudes in the process of diagnosis and treatment, informed consent and privacy protection are summarized in Table 2.

**Table 2:** Ethical attitude of medical staff in psychiatric staffs in Shanghai

**Elements**	**N**	**Completely disagree (%)**	**Disagree (%)**	**Moderate (%)**	**Agree (%)**	**Fully agreed (%)**	**Others (%)**
Guardian has authority over patient’s treatment decisions	1094	6.9	19.4	2.7	33.6	34.7	2.7
Compulsory treatment is/can be beneficial to patients	1094	1.8	10.0	2.4	34.1	51.1	0.6
More stringent standards are necessary in involuntary admissions	1094	2.3	6.5	2.7	24.6	63.9	0
Fully process in involuntary admission (intramuscular medication, etc.).	1094	2.5	4.5	1.7	17.1	73.9	0.3
Patients have rights to refuse medical treatment	1094	41.2	39.3	1.2	14.5	3.7	0
Doctors can record less stigmatized diagnosis	1094	31.3	24.5	3.3	27.4	13.5	0
Patients must sign written informed consent forms before participation in clinical trials	1094	3.7	11.6	2.3	22.3	60.1	0
Patient’s participation in clinical trials is doctor’s decision	1092	48.3	40.5	1.3	6.3	3.5	0
Patient’s participation in clinical trials is guardian’s decision	1094	23.5	49.6	3.3	17.0	6.6	0
Patients or his guardian must sign written informed consent form before participation in clinical trials	1094	3.0	6.7	2.5	23.9	63.9	0
Patients damaged in clinical trials should get free treatment	1094	2.8	5.6	2.3	20.9	68.4	0
Oral consent is sufficient for patient participation in clinical trials	1093	66.0	23.8	2.7	5.3	2.1	0
Biomedical research approved by foreign institutions need not be reviewed again in China	1094	62.3	26.4	5.4	4.3	1.6	0
Doctors can refuse to disclose specific drug types or names during clinical trials	1094	40.9	30.7	5.2	14.6	8.6	0
Medical certifications should have time limits	1092	10.0	29.7	6.4	31.4	22.3	0
Written applications must be submitted in requests for medical certifications	1094	2.8	7.2	4.7	30.7	54.4	0.2
Medical certification has no legal effect in case of judicial appraisal in civil disputes or criminal cases; patients with mental disorders require forensic psychiatry	1094	4.0	16.2	3.5	26.9	49.2	0.3
Judicial officers must obtain patient or guardian consent before copying medical information	1094	12.9	20.0	4.2	25.6	37.0	0.3
Doctor has the right to refuse to write medical certifications for patients	1093	31.9	33.6	2.6	14.4	16.5	1.0
Doctor has the right to prescribe hypnotics to personal friends	1091	11.3	19.1	4.8	22.0	42.4	0

## Discussion

Although China has many psychiatric hospitals, they have limited facilities or are inadequately equipped [5], and do not have enough economic resources to offer all people with mental disorders the most appropriate treatment [13]. Indeed individuals with mental disorders are highly stigmatized in China [5], and the majority of people who have a mental disorder have never been treated for it [2]. It is therefore important that more psychiatric facilities and resources are developed in China, but it is equally important that the clinical practice and research that is conducted in such facilities is implemented in an ethical manner with the view to protecting patients' rights and wellbeing.

Medical Ethics Committees can improve the quality of scientific research [14], and also can protect the welfare of mental patients. On the other hand, research conducted in psychiatric hospitals without such committees has the potential to undermine the patient's medico-legal rights and to cause harm [11]. The present study suggests that most psychiatric hospitals in Shanghai have no Medical Ethics Committee; only 12.2% of participants said their psychiatric institutions have such a committee. This compares to 57.56% of general hospitals in Shanghai having established Hospital Ethics Committees [8]. Unsurprisingly then, almost 3/4 of medical staff at psychiatric hospitals think a Medical Ethics Committee should be set up at their hospital.

Medical professionals need to be trained in ethics and to continue ethical training to during their careers [15]. This is especially important for psychiatric staff as their patient groups are particularly vulnerable. It is particularly concerning then that continuing education in medical ethics is lacking and inadequate [16]. Most Chinese psychiatrists working in mental hospitals deal with patients with psychoses [17], the most vulnerable group of patients, but more than half the medical staff in our study admitted they had not received systematic education and training in medical ethics since commencing work. On the positive side, most of our participants wanted to increase their knowledge of ethics and ethical practices in the psychiatric setting.

Nearly all (97.2%) of the medical staff had not served as members of ethics committee that reviewed projects. This is unsurprising given the lack of such committees in the hospitals surveyed. Members of ethics committee should have a high level of knowledge about ethics [8], so the results of our study could reflect the real situation of medical staff in psychiatric hospitals. Less than 1/5 of our participants knew of the Nuremberg Code and the Declaration of Helsinki. More knew the local regulations relating to "biomedical research involving human subjects ethics review methods" and "drug clinical trial quality management practices". More interesting, nearly all participants (90.0%) knew the local law, the Shanghai Mental Health Regulations enacted by the Shanghai legislature in 2001. This was the first local regulation relating to mental illness in Mainland China. As yet, there is no national mental health law in China, despite two decades of effort [2]. We think that the establishment of a national mental health law would improve medical ethics knowledge in psychiatric institutions' staff [18].

This study also reveals some misconceptions held by medical staff in Shanghai’s psychiatric hospitals. About 41% of the participants agreed that although such action is contrary to under the Shanghai Mental Health Regulations, doctors sometimes change medical diagnosis certification in order to convey a less discriminatory diagnosis. This study also showed 40.9% and 30.7% of the participants completely disagree or disagree that doctors can refuse to tell the specific types or names of drugs in clinical trials, respectively. However, it is mandatory for the great majority of clinical trials to be randomized and double-blind in accordance with the Good Clinical Practice (GCP) certification program set up by the China State Food and Drug Administration. Other psychiatric clinical trials are required to be single blind at least [19]. More than half of participants (25.6% agree and 37.0% fully agree) thought that Judicial officers must obtain the consent of patients or their guardians before copying medical information, though this is not required by Shanghai Mental Health Regulations. The study showed that medical staff in Shanghai make a considerable effort to protect the privacy of patients with mental disorders. The patients have the right to get a medical certification, but still 14.4% and 16.5% of participants agree and fully agree that doctors could refuse writing certification of medical diagnosis. Of great concern is that 66% of participants agreed or strongly agreed that doctors could prescribe hypnotics to their friends, despite these medications being strictly limited prescription drugs rather than over-the-counter (OTC) medicines.

Communication plays a significant role in enhancing comprehension of consent information among patients [20]. However, this study also showed most of the staff had not discussed with patients or their guardians patients' participation in clinical trials. Some the staff did not want to interview patients to invite them to participate in clinical trials because they were worried about the possibility of poor outcomes and severe side effects. Most participants (78.6%) were not sure whether clinical trials help in the treatment of patients. About half of them (41.2%) thought that the clinical trials are initiated by pharmaceutical companies. These findings suggest that the medical staff who participated in this study do not know enough about clinical trials. Obviously, it is important to improve research ethics in China, whose endeavor to become a scientific superpower cannot be realized fully until a robust infrastructure of ethical review and strong governance is implemented and applied rigorously [16].

Despite some medical staff having misinformation regarding some aspects of ethics, the present study raises important practical and ethical considerations for health policy makers. Firstly, about half of participants considered the guardian could make a decision as to whether a patient could participate in a clinical trial or not, although more thought that it is better to get written informed consent from the patients themselves. Additional safeguards are needed in the informed consent process in psychiatric research [21]; medical staff in Shanghai’s psychiatric hospitals believe that this process could improve the patients' understanding of research. Secondly, a large majority of participants (85.2%) agreed with compulsory treatments. This suggests that medical staff in psychiatric hospitals in China are more inclined than not to treat patients in need. This may be due to the fact that in developing countries, including China, the focus of mental health policy is on severe psychosis [2], which emphasizes more stringent standards and involuntary admission due to the ‘do no harm’ principle [22]. Thirdly, the results showed that most psychiatric staff held that patients must sign written informed consent instead of relying on a decision made by doctors when they participated in clinical trials. Less than 10% (7.4%) of them think oral consent is sufficient if patients are willing to participate in clinical trials. Further, nearly 90% of medical staff think biomedical research which has been approved by foreign institutions needs to be reviewed again in China. Most of them considered that patients who are damaged in clinical trials should get free treatment.

Social changes over the past 25 years in China, especially in the eastern regions such as Shanghai, have gradually increased the recognition of the importance of mental health, and the pronounced economic improvements in China have allowed allocation of additional resources to mental health. However, there is still considerable work needed. Medical staff have little or no training in ethical of mental health practice and research, so they are unable (and often unwilling) to provide better psychiatric services [15]. Absence of knowledge about the ethics of mental illness and negative attitudes about the mentally ill prevent many sufferers from getting more psychiatric care. Though medical research ethics has been a growing issue in China over the past two decades [23], the knowledge of clinical research in mental health is poor, so researchers are unable to provide policy makers with useful information. However, the future looks promising. Evidence-based medicine is becoming more widespread and clinical trials are effectively monitored in China [24]. China’s clinical research ethics will become increasingly widespread and standardized [11].

### Study limitation

According to our original design, the questionnaires were self-completed; therefore, the findings of the survey may have some bias. A strength of our study was that we engaged a large sample from all of psychiatric hospitals in Shanghai, and our partition rate was 100%. However, the extent to which this sample is nationally representative is uncertain. Shanghai, is the most developed metropolitan area in China. It is unlikely that the results of this survey reflect the opinions of the whole nation. However, the goal of the present study was not to reach definitive conclusions about the knowledge of ethics in psychiatric institutions. The goal was to gather preliminary data on the attitude of medical staff. In our view, the present data suffice to show that the ethical issues related to clinical trials are insufficiently understood. If we do generalize our results to all of China, they would suggest that psychiatric staff should receive professional development in relation to ethics.

## Conclusions

This preliminary study provides the first data relating to the attitudes and knowledge of ethical research practices of medical staff at psychiatric hospitals in Shanghai. The results suggest the ethics knowledge is less than satisfactory. Information, understanding, and voluntary agreement are three key components of informed consent. However, existing practices related to informed consent do not adhere to required guidelines, and the reasons for this are complex. Better education of staff may improve their knowledge and encourage them to change their attitudes. We suggest that ethics should be included in training courses such as medical degrees, and there should be compulsory professional development courses for new staff.

## Ethics approval

This study was conducted with the approval of the Medical Ethics Committee of Shanghai Mental Health Center (No. 2010–29, IORG Number: IORG0002202; FWA Number: FWA00003065).

## Endnote

^a^ Such high response rates are not uncommon in China

## Competing interests

The authors declare that they have no competing interest.

## Authors’ contributions

YX (guarantor) had the initial ideas for the research and organized the distribution of questionnaires. HL developed the questionnaire and was the project manager. LS and JH contributed to data collection and analyses. LS, WY, HL, and YX provided training in the survey. All authors designed the study, interpreted the data, participated in writing the paper and approved the final draft.

## Funding

The study was made possible by the research funding provided by the Major National Special Fund (2008ZX09312-003) and Shanghai Medical Ethics Society Fund (2011–002).

## Pre-publication history

The pre-publication history for this paper can be accessed here:

http://www.biomedcentral.com/1472-6939/13/8/prepub
